# Restoration of skeletal muscle function via mesenchymal stem cells: mechanistic insights and therapeutic advances in myasthenia gravis

**DOI:** 10.3389/fcell.2025.1658062

**Published:** 2025-09-23

**Authors:** Xinyi Zhang, Dongmei Zhang, Ying Zhang, Jian Wang, Jing Lu

**Affiliations:** ^1^ College of Chinese Medicine, Changchun University of Chinese Medicine, Changchun, Jilin, China; ^2^ Scientific Research Office, The Affiliated Hospital to Changchun University of Chinese Medicine, Changchun, Jilin, China; ^3^ Department of Encephalopathy, The Affiliated Hospital to Changchun University of Chinese Medicine, Changchun, Jilin, China

**Keywords:** mesenchymal stem cells, skeletal muscle, myasthenia gravis, immunity, clinical translation

## Abstract

Mesenchymal stem cells (MSCs) have demonstrated distinct advantages in skeletal muscle repair owing to their self-renewal capacity, multidirectional differentiation potential, and immunomodulatory functions. As a critical regulator of skeletal muscle regeneration, MSCs have been shown to ameliorate skeletal muscle injury induced by factors such as wasting and metabolic disorders through the activation of satellite cell function, inhibition of myofiber atrophy, and regulation of protein metabolic balance. In the treatment of myasthenia gravis (MG), the therapeutic effects of MSCs are exerted through dual mechanisms: first, autoantibody production is reduced via immunomodulation, thereby alleviating immune-mediated attacks at neuromuscular junctions; second, secondary muscle atrophy is delayed by preserving the integrity of neuromuscular signaling. Notably, MSC function is closely associated with acetylcholine metabolism, neuromuscular junction stability, and the aging microenvironment, in which aging-induced MSC decline may exacerbate intramuscular fat infiltration and impair regenerative capacity. In this paper, the biological properties of mesenchymal stem cells (MSCs) and their regulatory roles in skeletal muscle metabolic and injury-related abnormalities are systematically reviewed, and the fundamental significance of MSCs in skeletal muscle repair and myasthenia gravis (MG) therapy is elucidated through multiple mechanisms, including immunomodulation, neuroprotection, and muscle fiber regeneration. Furthermore, the bottlenecks of clinical translation (including cell source selection, phenotypic stability, and efficacy heterogeneity) are analyzed, and the challenges and optimization strategies for clinical application are discussed, with the aim of providing theoretical references for regenerative medicine research in neuromuscular diseases. However, clinical translation studies have indicated that the actual efficacy of most MSC-based therapies is considerably lower than that observed in *in vitro* experiments. This discrepancy may be attributed to low post-transplantation cell survival, inadequate homing efficiency, and the adverse influence of a senescent microenvironment that impairs cellular function. It has been indicated by recent studies that strategies, including optimization of cell sources and preparation protocols (e.g., the use of allogeneic MSCs derived from adipose tissue or umbilical cord with standardized production), incorporation of biomaterial supports (such as hydrogel-based encapsulation), and adoption of combination therapies (e.g., co-administration with neurotrophic factors or targeted drugs), can effectively improve the delivery efficiency and therapeutic outcomes of MSCs.

## 1 Introduction

**FIGURE 1:**
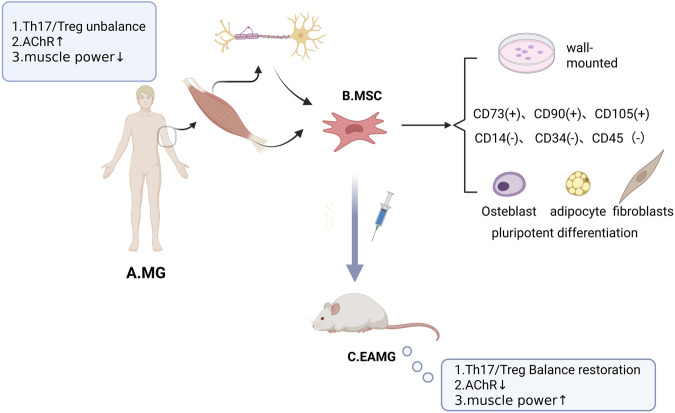
The Relationship between MSCs and MG. **(A)** Pathologic manifestations of MG include an imbalance in the Th17/Treg cell subset ratio, elevated serum anti-acetylcholine receptor (AChR) antibody titers, and decreased skeletal muscle strength. **(B)** First, skeletal muscle-derived MSCs are distributed at the muscle-ligament junction, motor nerve axons, and the periphery of the neuromuscular junction (NMJ). Secondly, MSCs were identified by 1. Plastic apposition properties when cultured *in vitro*; 2. Specific expression of surface markers such as CD73, CD90, and CD105, along with absence of hematopoietic markers (CD14, CD34, and CD45); and 3. Multipotent differentiation: osteoblastogenic, lipogenic, and fibrogenic. **(C)** Experimental autoimmune myasthenia gravis (EAMG) is an established animal model of MG. Administration of MSCs in EAMG exerts therapeutic effects, including restoration of the Th17/Treg cell subset ratio, reduction of serum anti-acetylcholine receptor (AChR) antibody titers, and improvement of skeletal muscle strength.

Myasthenia Gravis (MG) is an autoimmune disorder characterized by fluctuating skeletal muscle weakness and fatigability, with the hallmark feature of diurnal variation, in which symptoms are mild in the morning and worsen in the evening, aggravated by exertion and relieved by rest. The extraocular muscles are the most commonly affected, resulting in ptosis and diplopia. Other skeletal muscles throughout the body may also be involved, leading to facial weakness, difficulties in chewing and swallowing, dysarthria, head drop, and proximal limb weakness (e.g., difficulty in raising the arms or climbing stairs). In severe cases, respiratory muscle involvement can cause life-threatening dyspnea. Although traditional immunosuppressive therapies can partially alleviate symptoms, the development of interventions designed to restore skeletal muscle function remains limited. In recent years, mesenchymal stem cells (MSCs) have attracted increasing attention in myasthenia gravis (MG) therapy owing to their distinctive immunomodulatory properties and tissue repair capacity (Figure 1). The present study demonstrates that transplantation of MSCs in the experimental autoimmune myasthenia gravis (EAMG) model leads to a reversal of the Th17/Treg cell subset imbalance and a reduction in serum anti-acetylcholine receptor (AChR) antibody titers.

However, the precise mechanisms underlying the therapeutic effects of MSCs in MG, particularly the synergistic pathways involved in immunomodulation and skeletal muscle repair, remain insufficiently elucidated. To explore the association between MSCs, skeletal muscle and MG, in this paper we first systematically explain the biological properties of MSCs, including immunoregulation, multidirectional differentiation and paracrine function, and elucidate the theoretical basis for their therapeutic use. Then we analyze the interactions between MSCs and skeletal muscle, with an emphasis on their role in the regulation of metabolic homeostasis and injury repair. Then we systematically integrate and analyze the progress of the research on the treatment of MG with MSCs, and analyze the rescue effects of MSCs-mediated skeletal muscle regeneration, metabolic reprogramming and immune microenvironment remodeling on the pathological regression of MG. Finally, strategies to enhance the efficiency of MSC therapy are summarized, and multidimensional evidence-based guidance for clinical translation is provided. Based on the core pathological phenotype of MG skeletal muscle weakness, this paper delineates an integrated regulatory module of “stem cell-immune-muscle regeneration”, which aims to provide a roadmap for the subsequent mechanism analysis and clinical intervention.

## 2 Mesenchymal stem cell (MSCs)

### 2.1 Definition and multidirectional differentiation potential of MSCs

MSCs are a type of stem cells derived from the mesoderm and are widely distributed in tissues such as bone marrow, the umbilical cord, and the placenta. In addition to these tissues, MSCs are primarily localized in the perivascular region. Crisan and colleagues identified functional progenitor cell reservoirs within the vascular wall using tissue-specific markers (Crisan et al., 2008). Furthermore, their homing process was found to be significantly dependent on the vascular network.

MSCs possess multidirectional differentiation potential (Keating, 2006). Lineage-specific differentitation of MSCs is regulated by specific factors in the microenvironment, such as secreted Bone Morphogenetic Protein (BMP)9 to induce osteogenic differentiation (Mostafa et al., 2019).

### 2.2 Functionality and low immunogenicity of MSCs

**FIGURE 2:**
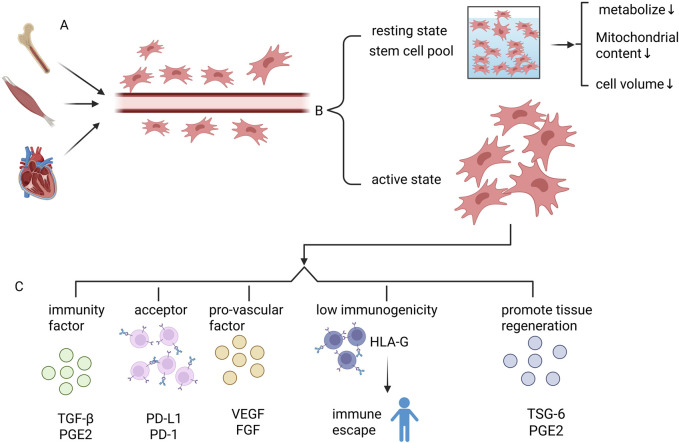
Basic characteristics of mesenchymal stem cells MSCs. **(A)** MSCs are stem cells that are widely found in a variety of tissues, such as bone marrow, skeletal muscle, heart, etc. MSCs are mainly localized in the perivascular region. **(B)** MSCs in the body are divided into two categories, one is active MSCs. The other constitutes the stem cell pool of MSCs, which maintains the quiescent state. MSCs in the stem cell pool have reduced metabolic levels, decreased mitochondrial content, and reduced cell size. **(C)** Active MSCs have the following roles: 1. Secretion of immune factors, such as TGF-β, PGE2; 2. Direct contact between membrane surface molecules and immune cells to mediate immunomodulation, such as PD-L1; 3. Secretion of pro-angiogenic factors (e.g., VEGF, FGF), to maintain homeostasis in the tissue microenvironment; 4. Low-immunogenicity: immune escape mechanism mediated by HLA-G molecules to Avoid host immune surveillance, significantly reducing the rejection reaction caused by allogeneic transplantation; 5. Secretion of TSG-6, PGE2 and other factors to promote tissue regeneration.

MSCs have multiple biological functions (Figure 2): Tissue microenvironmental homeostasis is maintained through the secretion of immunomodulatory factors (e.g., TGF-β, PGE2) (Kyurkchiev et al., 2014) and pro-angiogenic factors (e.g., VEGF, FGF) in a synergistic manner (Krakora et al., 2013). At the same time, metabolic disorders can be alleviated through regulation of the insulin signaling pathway and key genes involved in glucose and lipid metabolism (e.g., GLUT4, PPARγ). MSCs can improve the metabolic disorder status (Ji et al., 2015). These multidimensional regulatory capabilities endow them with unique therapeutic potential in immune repair, angiogenesis and metabolic imbalance intervention.

MSCs are also characterized by low immunogenicity and are capable of evading host immune surveillance through HLA-G–mediated immune escape mechanisms, thereby significantly reducing allograft rejection (Park et al., 2024). Its functional realization relies heavily on the dual modes of action: ① Immunomodulation through direct contact with immune cells via molecules such as PD-L1 on the membrane surface; ②Secretion of paracrine factors such as TSG-6 and PGE2 which are involved in inflammation regulation and tissue regeneration. Notably, recent studies have found that MSCs can also activate skeletal muscle satellite cells by secreting IGF-1, which significantly promotes muscle regeneration and injury repair (Baht et al., 2020).

### 2.3 Criteria for isolation and characterization of MSCs

As basic research and clinical translation of MSCs continue to advance, the lack of standardization in isolation and culture systems, phenotypic identification standards, and functional validation methods has become bottlenecks restricting widespread clinical application. In this context, the International Society for Cellular Therapy (ISCT) established criteria for the identification of MSCs in 2006, which explicitly require that the following core characteristics be met (Orbay et al., 2012): i Adherence to plastic surfaces under *in vitro* culture conditions; ii Specific expression of surface markers including CD73, CD90, and CD105, with the simultaneous absence of hematopoietic markers (CD14, CD34, CD45) and MHC class II molecules; iii Potential to differentiate into mesodermal cells (osteogenic, lipogenic, chondrogenic and myogenic). During the isolation and purification of MSCs, researchers can screen for lineage-specific surface markers such as Platelet-Derived Growth Factor Receptor α (PDGFRA) and Stem Cell Antigen-1 (Sca-1), and combine the above molecular characteristics in Fluorescence-Activated Cell Sorting (FACS) or Magnetic-Activated Cell Sorting (MACS) to obtain high-purity MSC populations (Uezumi et al., 2010).

### 2.4 Biological characterization of resting MSCs

The maintenance of a quiescent state by MSCs in tissues such as skeletal muscle, bone and adipose tissue is a central mechanism for the long-term preservation of their stem cell pool. This quiescent state represents a reversible, long-term exit from the cell cycle that serves dual functions in maintaining tissue homeostasis: supporting circulating progenitor cells and acting as a strategic reserve for tissue repair. Notably, quiescent MSCs exhibit remarkable self-renewal potential, a characteristic that declines rapidly during *ex vivo* culture but is restored following *in vivo* transplantation. This dynamic plasticity underscores a close link between the maintenance of stemness and regulation by the microenvironment (de Morree and Rando, 2023).

Genetic studies have shown that the quiescent state of MSCs requires the active participation of multiple signaling pathways. Once these pathways are disturbed, stem cells can be prematurely activated, which results in the loss of self-renewal capacity and ultimately leads to depletion of the stem cell reservoir and reduced regenerative potential *in vivo*, thereby accelerating the aging process.

For example, the balance between proliferation and differentiation of MSCs can be regulated, at least in part, through the Wnt/β-catenin axis. Activation of this axis has been shown to induce MSCs to upregulate the expression of MyoD, myogenin, and other myogenic regulators, thereby promoting myogenic differentiation (Zhou et al., 2015).

Resting-state MSCs exhibit several unique cellular properties: a smaller cell volume (10–15 μm in diameter), an approximately 60% reduction in basal metabolic rate, a decrease in mitochondrial content to 30%–40% of that in proliferating cells, and a marked downregulation of RNA and protein synthesis rates were observed. This metabolic dormancy is associated with three biological advantages: enhanced resistance to cellular stress, preservation of genomic stability, and the establishment of phenotypic compartmentalization from activated descendant cells. Upon tissue injury, an activation program is rapidly initiated by these cells, the G0–G1 transition is completed within 24–48 h, and both tissue repair and the stem cell pool are regenerated through asymmetric division (de Morree and Rando, 2023).

MSCs have been shown to significantly enhance muscle repair through paracrine mechanisms, both *in vitro* and *in vivo* (Chiu et al., 2020). However, a clear distinction in surface markers can be observed between muscle stem cells (MuSCs, i.e., satellite cells) and mesenchymal stem cells (MSCs): MuSCs are characterized by the expression of VCAM-1, while MSC markers such as Sca-1 are generally absent (Liu et al., 2015). Despite differences in origin and phenotype, both cell types are considered to play crucial roles in skeletal muscle repair and are thought to partially share regulatory mechanisms governing quiescence and activation. Under physiological conditions, both MuSCs and MSCs are predominantly maintained in a quiescent state. Following skeletal muscle injury, factors such as inflammatory signals within the microenvironment are capable of triggering the activation and proliferation of these cells. Once activated, MuSCs primarily undergo proliferation and are subsequently directed to differentiate into myoblasts, which then fuse to form new muscle fibers or repair damaged ones, thereby acting as the principal executors of muscle regeneration. In contrast, activated MSCs contribute primarily through paracrine actions (i.e., secretion of various cytokines and growth factors) and immunomodulatory functions, thereby establishing a favorable microenvironment that facilitates the proliferation and differentiation of MuSCs and indirectly promotes regeneration (Theret et al., 2021; Molina et al., 2021). Additionally, MSCs are also capable of differentiating into other mesenchymal lineage cells, including fibroblasts and adipocytes. For example, studies have indicated that MSCs may influence the regeneration process through the secretion of specific extracellular vesicles (e.g., MSCs-ApoEVs) (Ye et al., 2023). In the later stages of repair, the majority of activated MuSCs are differentiated and consumed, with only a small number undergoing self-renewal and subsequently returning to a quiescent state to replenish the stem cell pool (Fu et al., 2022); meanwhile, activated MSCs are thought to contribute to the reestablishment of tissue homeostasis through differentiation or apoptosis.

### 2.5 Spectral association and functional heterogeneity of mesenchymal stem cell (MSCs), mesenchymal progenitor cell (MPCs) and fibro/adipogenic progenitors (FAPs)

There is often confusion in the classification of Mesenchymal Stem Cells (MSCs), Mesenchymal Progenitor Cells (MPCs), and fibro/adipogenic progenitors (FAPs) in existing studies. It is widely recognized that MPCs represent the early developmental stage of MSCs, in which the former are progenitor cells characterized by high proliferative capacity, whereas the latter are mature stem cells possessing multidirectional differentiation potential. Although the two terms are often used interchangeably in the literature, they essentially represent distinct differentiation states within the same cell lineage (Sarugaser et al., 2009).

Fibro/Adipogenic Progenitors (FAPs) are considered a tissue-specific population of mesenchymal stem/progenitor cells residing in skeletal muscle. They share multiple characteristics with the extensively studied bone marrow–derived mesenchymal stem cells (MSCs), including adherent growth *in vitro*, expression of similar surface markers (such as PDGFRα^+^ and Sca-1^+^), and the potential to differentiate into fibroblasts and adipocytes (Kim and Braun, 2020; Uezumi, 2023). However, FAPs are specifically localized within the interstitial spaces of skeletal muscle, and their functions and behaviors are strongly regulated by the muscle microenvironment. In healthy muscle, they are primarily involved in maintaining extracellular matrix homeostasis. Following injury, they are rapidly activated and transiently contribute to the regenerative function of muscle stem cells (MuSCs) through paracrine signaling, before being cleared via apoptosis. Dysregulation of their fate may result in pathological fibrosis or fatty infiltration (Joe et al., 2010).

Therefore, although homology is exhibited in the *in vitro* identification criteria, FAPs should be more accurately described as muscle-resident MSC-like cells characterized by distinct tissue localization and specialized functions, rather than being merely equated with bone marrow MSCs.

## 3 MSCs and skeletal muscle

This section first examines the physiological state of MSCs in skeletal muscle and then highlights their significance in skeletal muscle injury and metabolic disorders.

### 3.1 MSCs and satellite cell

Two types of stem cell subpopulations are present in the skeletal muscle microenvironment: satellite cells (muscle stem cells, or MuSCs) and MSCs with myogenic differentiation potential. Satellite cells are crucial progenitor cells involved in skeletal muscle repair. During muscle regeneration, MSCs are considered to play a pivotal role in modulating the activation, proliferation, and differentiation of satellite cells. This regulatory mechanism is regarded as essential for the restoration of functional muscle tissue. Specifically, local injection of MSCs at the injury site has been shown to significantly enhance the proliferation and differentiation of satellite cells (MuSCs) via the secretion of bioactive factors, thereby facilitating the targeted regeneration of skeletal muscle fibers and the subsequent formation of functional muscle tissue (Sandonà et al., 2021).

### 3.2 Ecological niche of MSCs in skeletal muscle

The anatomical localization of myosatellite cells and myogenic MSCs differs markedly. Satellite cells, as skeletal muscle-specific stem cells, are strictly localized in the anatomical gap between the basal layer of muscle fibers and the myofibrillar membrane, whereas MSCs are primarily localized in the interstitial region and exhibit high-density aggregation at the muscle-ligament junction (Uezumi et al., 2010; Uezumi et al., 2021). Notably, subpopulations of MSCs exhibited a distinct tendency toward regionalized distribution. Some cells were localized in close proximity to motor nerve axons, whereas others selectively encircled the periphery of the neuromuscular junction (NMJ). This spatial specificity suggests that they may participate in regulating NMJ function through physical proximity or paracrine signaling. Based on this spatial distribution property, it was concluded that MSCs constitute essential cellular components for maintaining the structural integrity of the NMJ and muscle mass (Kurosawa et al., 2021). It was experimentally confirmed that transplantation of exogenous MSCs can improve neuromuscular conduction function by promoting nerve axon regeneration and NMJ structural remodeling (Kim et al., 2012).

### 3.3 Skeletal muscle and metabolic diseases

Skeletal muscle abnormalities have been associated with metabolic diseases. Skeletal muscle, as the largest metabolic regulatory organ in the human body, is responsible for maintaining metabolic homeostasis through the regulation of substrate utilization processes, including glucose and fatty acid uptake and oxidation. Under conditions of elevated insulin levels or physical activity, glucose uptake and utilization are enhanced in skeletal muscle; whereas during prolonged exercise or fasting, energy production is more dependent on fatty acid oxidation (Fan et al., 2024). Such adaptive regulation of substrate utilization is essential for the maintenance of blood glucose stability, lipid balance, and overall energy metabolism. Pathological remodeling of skeletal muscle can be induced by metabolic disorders such as obesity, aging, and diabetes (Nunan et al., 2022). The balance of energy metabolism in skeletal muscle cells is disrupted by these metabolic disorders through the activation of excessive mitochondrial fission pathways, which consequently inhibit the proliferative potential of stem cells and their capacity for tissue repair (Hong et al., 2022; Jheng et al., 2012). Notably, ectopic deposition of intramuscular adipose tissue has been reported to be positively associated with the development of obesity, which in turn exacerbates skeletal muscle dysfunction through chronic inflammation and lipotoxicity, thereby establishing a vicious cycle of metabolic disturbances (Wang et al., 2024).

#### 3.3.1 Metabolic diseases and abnormal differentiation of MSCs

**FIGURE 3:**
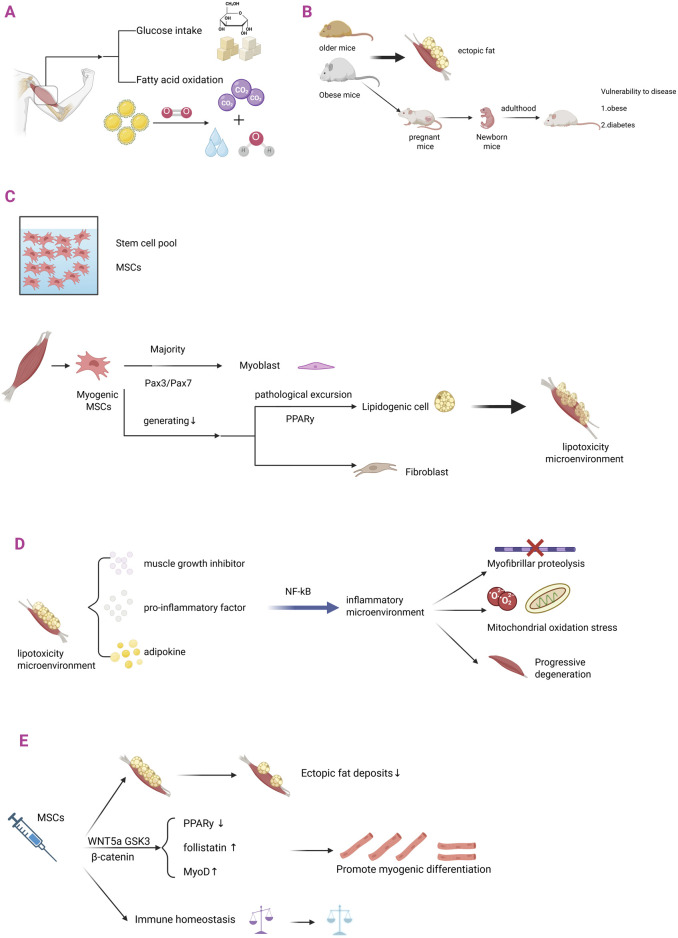
MSCs in ectopic fat infiltration in skeletal muscle. **(A)** Skeletal muscle is the largest metabolic regulatory organ in the human body and is a major functional site for glucose uptake and fatty acid oxidation. **(B)** The differentiation pathway of skeletal muscle-derived MSCs is: most of them differentiate into myoblasts through the Pax3/Pax7 pathway; a small portion undergoes lipogenic differentiation and fibrogenic differentiation. **(C)** Obese and aged laboratory mice are prone to ectopic fat infiltration in skeletal muscle. Metabolic abnormalities in obese female mice can affect their foetuses, increasing the risk of metabolic diseases such as diabetes and obesity in adulthood. **(D)** When metabolic diseases occur, the differentiation of myogenic MSCs is pathologically shifted: there is a decrease in the amount of myogenic differentiation; there is an increase in the amount of lipogenic differentiation (mainly through the PPARy pathway); this phenomenon leads to a lipotoxic microenvironment in skeletal muscle. **(E)** The lipotoxic microenvironment in skeletal muscle, which is characterized by a pathological cascade of muscle growth inhibitor (Myostatin), pro-inflammatory factors, and adipokines (leptin/Leptin). These pathological factors establish a chronic low-grade inflammatory microenvironment in skeletal muscle through the activation of classical inflammatory pathways such as NF-κB, triggering myofibrillar protein catabolism, mitochondrial oxidative phosphorylation dysfunction, and ultimately leading to progressive degradation of skeletal muscle contractile function. **(F)** When MSCs are injected to treat metabolic diseases: (i) ectopic fat deposition rate decreases; the WNT5a/GSK3/β-catenin signaling axis balances the direction of MSCs differentiation through spectrum-specific regulation - not only inhibiting the activity of PPARγ (a key lipid-forming transcription factor) through phosphorylation, but also activating follicle suppressor expression; (ii) upregulation of MyoD myogenic factors to promote myogenic differentiation; and (iii) restoration of immune homeostatic balance.

A significant pathological association has been identified between metabolic diseases and abnormal differentiation of MSCs (Figure 3). MSCs exhibited the highest cell density during fetal and infantile stages. Their mitochondrial oxidative phosphorylation activity was found to be significantly correlated with the maternal metabolic microenvironment, indicating that maternal-derived metabolic signals play a dominant role in regulating stem cell metabolism during fetal stages (McCurdy et al., 2016). As individuals mature, MSCs progressively decline through directional differentiation, and their metabolic activity in adulthood is predominantly regulated by exogenous factors, particularly nutritional intake patterns and mechanomechanical stimuli (e.g., dietary composition and exercise loads), which exert a critical influence on metabolic pathways. This temporal transition from “maternal-dependent metabolic programming” to “environmentally-adapted metabolic regulation” represents a central feature of the developmental heterogeneity of metabolic functions in MSCs (Erickson et al., 2021; Akhaphong et al., 2021).

#### 3.3.2 Abnormal differentiation of MSCs and skeletal muscle

The metabolic adaptations of skeletal muscle are closely associated with the determination of the differentiation fate of intrinsic MSCs. During physiological skeletal muscle development, the majority of myogenic MSCs, except for quiescent MSCs, initiate a myogenically directed differentiation program. A PAX3/PAX7-positive progenitor cell stage is undergone, followed by differentiation into functional myoblasts characterized by the expression of myogenic regulatory factors (MRFs) (Maroto et al., 1997). The remaining cells are directed to differentiate into adipocyte and fibroblast lineages. When the metabolic pathway associated with the myogenic differentiation of MSCs is adaptively impaired, the efficiency of their myogenic differentiation is markedly reduced, leading to a lineage shift toward preferential differentiation into preadipocytes. These precursor cells subsequently mature into adipocytes through the progressive accumulation of intracellular lipid droplets (Wosczyna et al., 2021), ultimately resulting in an ectopic fat deposition pathological phenotype.

When the above pathological processes occur during maternal gestation, the maternal obesity-induced pathological metabolic microenvironment is associated with a shift in the spectral differentiation of fetal MSCs. This manifestation is characterized by impaired myofibrillar production, a reduced number of myofibers, and a decreased cross-sectional area. This process is further accompanied by ectopic infiltration of intramuscular adipose tissue and an increase in interstitial fibrosis (Yan et al., 2011).

Maternally derived metabolic disorders have been recognized as significant risk factors for offspring who develop metabolic susceptibility to type 2 diabetes and obesity in adulthood (Samuelsson et al., 2008). The central mechanism is believed to involve maternal metabolic disorders that programmatically influence the metabolic phenotype in later life through the induction of impaired mitochondrial biosynthesis in fetal skeletal muscle. This impairment is characterized by reduced mitochondrial density and diminished activity of the oxidative phosphorylation complex, accompanied by compensatory upregulation of fatty acid oxidation pathways and inhibition of glucose metabolism (Du et al., 2010). Skeletal muscle, as the largest insulin-sensitive tissue in the body, together with its pathological fat deposition, has been shown to directly impair insulin signaling and is considered a key mechanism in the pathogenesis of type 2 diabetes mellitus (Aguiari et al., 2008).

In contrast, the therapeutic potential of MSCs in diabetes has been attributed to their ability to modulate the expression of critical metabolic regulators within skeletal muscle tissues. Systemic transplantation of MSCs has been demonstrated to specifically upregulate the expression of interleukin-1 receptor antagonist (IL-1Ra) and glucose transporter protein 4 (GLUT4) within the skeletal muscle microenvironment, thereby improving glucose homeostasis in diabetic animal models through the suppression of local inflammatory responses and the enhancement of insulin sensitivity (Ji et al., 2015).

#### 3.3.3 Ectopic fat deposits in skeletal muscle

Ectopic fat deposition in the skeletal muscle microenvironment is caused by an imbalance in the lineage differentiation of MSCs. When the PPARγ signaling pathway is pathologically activated, MSCs are driven toward adipogenic differentiation, resulting in abnormal proliferation of adipose precursor cells and excessive formation of mature adipocytes, which together form the pathological basis of ectopic fat deposition (Grimaldi, 2001).

This aberrant adipogenic process is manifested through the induction of a lipotoxic microenvironment in skeletal muscle, characterized by a pathological cascade involving Myostatin, pro-inflammatory factors (IL-1β, IL-6, IL-8, TNF-α), and adipokines (leptin). These pathological factors contribute to the establishment of a chronic low-grade inflammatory microenvironment in skeletal muscle via activation of classical inflammatory pathways, such as NF-κB, thereby triggering myofibrillar protein catabolic hypermetabolism and impairing mitochondrial oxidative phosphorylation. This cascade ultimately results in the progressive degradation of skeletal muscle contractile function and the manifestation of myasthenic phenotypes (Cai et al., 2004).

Ectopic fat deposition within the skeletal muscle microenvironment constitutes a critical pathological basis for sarcopenia and is believed to exacerbate skeletal muscle dysfunction through dual mechanisms: first, ectopic fat accumulation in insulin-sensitive tissues, such as skeletal muscle, is thought to directly suppress the proliferation and differentiation of myogenic stem cells; second, the lipotoxic metabolic milieu accelerates muscle atrophy, thereby contributing to the establishment of a cascading pathological network described as “fat infiltration–myasthenia.” This cascading pathological network, referred to as “adipose infiltration–muscle atrophy,” further characterizes the degenerative process (Fan et al., 2024). Myasthenia gravis, a central phenotype of aging-related degenerative pathology, is characterized by progressive loss of skeletal muscle mass, reduced muscle fiber cross-sectional area, and a dynamic decline in muscle strength. Moreover, ectopic adipose infiltration is considered to significantly aggravate this degenerative process by disrupting the balance between myogenesis and catabolism (Fan et al., 2024).

#### 3.3.4 MSCs therapy for skeletal muscle metabolic diseases

The significant efficacy of MSCs in treating metabolic disorders of skeletal muscle has been demonstrated in recent studies. The core therapeutic value of MSCs is manifested in their microenvironment-dependent, multidimensional reparative functions:

First, the administration of MSCs was found to effectively reverse the fatty pathological infiltration of skeletal muscle. Preclinical studies have demonstrated that localized MSC transplantation during tendon repair can improve pathological outcomes through two regulatory mechanisms: 1 specific inhibition of ectopic fat deposition within the injured rotator cuff muscles, and 2 upregulation of Tenascin-C, a core protein of basement membrane structure, synergistically optimizes the spatial conformation of extracellular matrix (ECM) and effectively blocks degenerative pathological remodeling of skeletal muscle and adipose transdifferentiation (Flück et al., 2021). Secondly, MSCs treatment was found to enhance muscle regeneration potential by significantly promoting myogenic differentiation efficiency, thereby inhibiting ectopic fat invasion. Molecular mechanistic studies confirmed that the WNT5a/GSK3/β-catenin signaling axis balances the direction of MSCs differentiation through spectrum-specific regulation - not only inhibiting PPAR-γ (key lipogenic transcription factor) activity through phosphorylation, but also activating follicle suppressor expression. As a core regulator of myogenic differentiation, follistatin promotes myogenic lineage-directed differentiation of MSCs by antagonizing myostatin signaling, thereby enhancing muscle regeneration efficacy (Reggio et al., 2020). Fibroblast growth factor 8b (FGF8b), which is dynamically upregulated during tissue repair, has been identified as a critical regulatory molecule in the fate determination of MSCs. FGF8b specifically inhibits the C/EBPα-mediated adipogenic differentiation program via activation of the MAPK/ERK signaling pathway, while simultaneously enhancing the expression of MyoD and other myogenic regulatory factors, thereby promoting the predominance of myogenesis during tissue repair (Otsuka et al., 2024). This mechanism suggests that the endogenous microenvironment dynamically regulates the lineage specification of MSCs through a precise network of factors, thereby offering a novel target for therapeutic intervention in regenerative medicine.

Therapies based on mesenchymal stem cells (MSCs) have demonstrated considerable potential in the treatment of sarcopenia. It has been reported that human umbilical cord–derived mesenchymal stem cells (hUC-MSCs) mitigate the pathological progression through several regulatory mechanisms, including remodeling of the extracellular matrix (e.g., upregulation of laminin and collagen IV), activation of the myosatellite cell regeneration program, enhancement of autophagic flux to eliminate damaged organelles, and inhibition of senescence-associated secretory phenotype (SASP)-mediated pro-atrophic cascade responses, thereby preventing the pathological pathway leading to progressive muscle mass loss (Wang et al., 2023).

### 3.4 Skeletal muscle disorders

Skeletal muscle mass loss is commonly observed in pathological states such as aging, chronic diseases, and autoimmune disorders. Although skeletal muscle has the potential for endogenous regeneration, which can compensate for approximately 20% of muscle mass loss (Liu et al., 2018), this capacity is limited. When the injury exceeds this threshold, pathological outcomes, including progressive loss of muscle strength and motor dysfunction, are likely to occur. The process of skeletal muscle regeneration is composed of two sequential phases: a degenerative phase, defined by myofiber necrosis and infiltration of inflammatory cells, and a regenerative phase, during which satellite cells differentiate into multinucleated myotubes that subsequently form new myofibers. Quantification of Central Nucleated Fibers (CNFs) serves as a key indicator in the assessment of the regenerative process (Iyer et al., 2020). Furthermore, the restoration of innervation is essential for functional reconstruction (Chargé and Rudnicki, 2004).

MSCs, as adult stem cells with multidirectional differentiation potential, have been widely applied in regenerative medicine (Chiu et al., 2020). They are particularly effective in promoting the repair of the bone and skeletal muscle systems, as well as in ligament and tendon regeneration (Park et al., 2024; Chamberlain et al., 2017).

#### 3.4.1 MSCs and skeletal muscle injury--inflammatory cell infiltration stage

Skeletal muscle injuries are characterized by muscle fiber necrosis, which subsequently triggers an inflammatory response. Innate immune cells, including macrophages and neutrophils, are rapidly recruited to the injury site (Forcina et al., 2020). In addition to removing necrotic tissue and cellular debris, these immune cells secrete various cytokines, including IL-6 and TNF-α (Saito et al., 2020). These factors promote skeletal muscle regeneration through mechanisms that involve the synergistic modulation of inflammatory responses and the activation of repair-associated cells.

**FIGURE 4:**
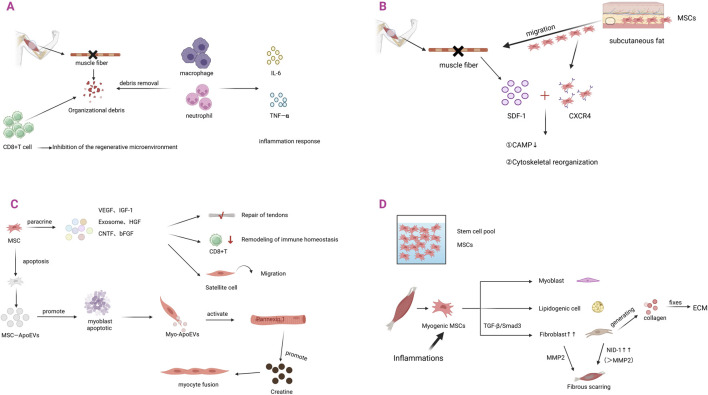
The role of MSCs in skeletal muscle injury and regeneration. **(A)** Skeletal muscle injury produces tissue debris. Macrophages and neutrophils remove tissue debris and secrete IL-6, TNF-α, leading to the development of inflammatory response. The increase of CD8^+^ T cells inhibits the microenvironment of regeneration. **(B)** After skeletal muscle injury, tissues release SDF-1, which triggers a stem cell homing response. MSCs in subcutaneous fat migrate to the site of injury occurrence and upregulate the expression of the receptor CXCR4. When CXCR4 binds to SDF-1, it firstly activates CAMP and secondly regulates cytoskeletal reorganization. **(C)** After homing to the site of skeletal muscle injury, MSCs primarily exert their effects through paracrine mechanisms. They can secrete VEGF, IGF-1, exosomes, HGF, CNTF, and bFGF. These factors promote tendon repair, remodel immune homeostasis, and facilitate satellite cell migration. When MSCs undergo apoptosis, they produce MSCs-ApoEVs, which can induce myogenic fusion. **(D)** Under inflammatory responses, MSCs undergo pathological shifts. Through TGF-β/Smad3 signaling, MSCs differentiate into fibroblasts, leading to their increased numbers. An appropriate amount of fibroblasts can secrete collagen, maintain ECM homeostasis, and promote skeletal muscle repair. Excess fibroblasts, on the other hand, lead to excessive accumulation of fibrillar collagens and fibronectin and formation of scarring, which molecularly manifests as overexpression of NID-1.

Mesenchymal stem cells (MSCs) are recognized as playing an important role in the remodeling of immune homeostasis (Figure 4). It has been demonstrated that, during the inflammatory response, pathological accumulation of CD8^+^ effector memory T (TEM) cells inhibits the regenerative microenvironment and delays the repair process. Moreover, immune homeostasis is remodeled by MSCs to promote tissue healing through regulation of the CD8^+^ TEM cell subset ratio (Ebner et al., 2020).

#### 3.4.2 Skeletal muscle repair process--directed migration of MSCs

The directional migration ability of MSCs represents a fundamental biological property that contributes to tissue repair. Studies have demonstrated that damaged skeletal muscle is specifically enriched in CD201^+^PDGFRA^+^ mesenchymal stromal cell populations of non-muscle origin, which exhibit a pathological expansion (Di Pietro et al., 2022). Notably, this amplification was not derived from *in situ* proliferation but was associated with a marked reduction in the number of MSCs within subcutaneous adipose tissue (Sastourné-Arrey et al., 2023). This phenomenon suggests that the injured microenvironment induces directional migration of adipose-derived MSCs across the tissue barrier to the injured region by releasing chemokines such as stromal cell-derived factor-1 (SDF-1). This migration mechanism highlights the pivotal role of MSCs in organismal self-repair. Through their intrinsic chemotactic properties, MSCs can detect pathological signals autonomously and precisely home to the site of injury, thereby initiating the muscle regeneration program without the need for external intervention.

When skeletal muscle is damaged, a chemotactic gradient is established primarily through the upregulation of stromal cell-derived factor-1 (SDF-1), which subsequently recruits MSCs to migrate to the injury site. The molecular mechanisms of SDF-1 involve remodeling of the actin cytoskeleton, thereby promoting the directional migration of MSCs through the activation of adhesion patch kinase, cell division cycle protein 42 (CDC42), and Ras-associated C3 botulinum toxin substrate 1 (Rac1) (Kowalski et al., 2017).

MSCs dynamically upregulate the expression of the CXCR4 receptor in response to injury signals by sensing the chemotactic gradient of SDF-1 (Sordi et al., 2005). Mechanistic studies revealed that SDF-1 binding to CXCR4 triggered cascading signaling events: on the one hand, it inhibited adenylate cyclase-mediated cAMP signaling (Stoicov et al., 2013), while regulating cytoskeletal reorganization through phosphatidylinositol 3-kinase (PI3K) and Rho- GTPases; on the other hand, it synergistically regulated ERK1/2 and p38 mitogen-activated protein kinase (MAPK) pathways, which together determined the MSCs migratory fate (Kasprzycka et al., 2019).

#### 3.4.3 Skeletal muscle repair process--repair mechanism of MSCs

The reparative effects of MSCs are largely mediated through their paracrine signaling network. This signaling network plays a pivotal role in bone and skeletal muscle regeneration, as well as in ligament and tendon repair, by secreting pleiotropic factors such as Vascular Endothelial Growth Factor (VEGF), Insulin-like Growth Factor-1 (IGF-1), Hepatocyte Growth Factor (HGF), Ciliary Neurotrophic Factor (CNTF), and basic Fibroblast Growth Factor (bFGF) (Borselli et al., 2010; Jiang et al., 2010). Preclinical studies have demonstrated that the local injection of mesenchymal stem cells (MSCs) significantly promotes the regeneration of muscle fibers in injured skeletal muscle. Key experimental evidence has confirmed that PKH26-labeled bone marrow-derived MSCs (BMMSCs) were eliminated by the host immune system within 21 days post-transplantation and did not directly participate in myotube fusion or myofiber formation; However, their paracrine effects persisted for more than 4 weeks. This phenomenon clearly indicates that the therapeutic effects of MSCs are mediated by a secretome-driven, non-cell-autonomous regulatory mechanism, rather than by the differentiation or long-term retention of the transplanted cells (Chiu et al., 2020). It has been further suggested that their therapeutic effects derive from a secretome-mediated molecular regulatory mechanism.

Apoptotic extracellular vesicles derived from MSCs (MSCs-ApoEVs) have been shown to possess distinct therapeutic potential in skeletal muscle repair. Studies have shown that MSCs-ApoEVs promote tissue repair through a dual regulatory mechanism: on the one hand, they enhance the immune-mediated clearance of damaged myofibers; on the other hand, they promote myogenic differentiation and fusion, thereby improving regenerative efficiency (Wang et al., 2025). The repair mechanism involves a cascade of regulatory processes: MSCs-ApoEVs induce programmed death of myoblasts by triggering the release of secondary apoptotic vesicles (Myo-ApoEVs), which specifically activate the Pannexin 1 membrane channel to mediate creatine efflux, whereas extracellular creatine serves as a key signaling molecule to promote myoblasts fusion. *In vitro* experiments demonstrated a significant increase in the cell fusion index following the treatment of C2C12 myoblasts with MSCs-ApoEVs (Ye et al., 2023).

Exosomes derived from MSCs have been shown to exhibit considerable therapeutic potential in skeletal muscle repair. Exosomes derived from MSCs are considered to play a critical role in this process through modulation of the inflammatory response, inhibition of fibrotic progression, and activation of myogenic pathways (Iyer et al., 2020). Among these, human umbilical cord-derived exosomes (UMSC-Exo) have been demonstrated to markedly enhance tissue regeneration following ischemic injury by delivering circHIPK3 non-coding RNA, which specifically suppresses pyroptosis in skeletal muscle cells (Yan et al., 2020).

The androgen receptor (AR) expressed on MSCs is considered to play a key role in skeletal muscle injury repair via genomic regulatory mechanisms. It has been demonstrated that AR binds to the androgen response element (ARE) in the promoter region of the Igf1 gene, and the presence of an H3K4me3 modification peak upstream of the transcription start site provides evidence that AR directly regulates Igf1 transcription, a mechanism resembling AR regulation of extracellular matrix (ECM) remodeling-related genes. Activated AR was found to induce MSCs to secrete IGF1, thereby promoting satellite cell activation and protein synthesis in a paracrine manner, while maintaining a balance between pro-regenerative factors (e.g., VEGF and HGF) and anti-fibrotic factors (e.g., MMP2). However, in the absence of androgen receptor (AR) signaling, MSCs shift toward a pro-fibrotic/adipogenic phenotype, leading to marked perineal muscle atrophy independent of age, accompanied by activation of cell death pathways and dysregulated expression of ECM-related genes. These alterations further exacerbate post-injury fat infiltration and ECM degradation. Moreover, local administration of IGF1 rescues AR deficiency–induced muscle atrophy, establishing IGF1 as a key downstream effector of AR-regulated MSCs and providing a novel therapeutic strategy for muscle atrophic disorders (Sakai et al., 2024).

Studies have further revealed that the WISP1 protein, which is secreted by MSCs, plays a critical role in muscle regeneration through activation of the Akt/mTOR pathway. Its expression declines markedly with aging, thereby serving as an important molecular marker of reduced regenerative capacity (Lukjanenko et al., 2019).

#### 3.4.4 Skeletal muscle repair complete - fibrous scar formation

The central hallmark of skeletal muscle repair lies in the dynamic equilibrium between muscle fiber regeneration and connective tissue reconstruction, a process that is often accompanied by fibrous scar formation. Fibrous scar formation primarily results from the activity of fibroblasts. Fibroblasts play a dual role in skeletal muscle repair through the synthesis of extracellular matrix (ECM): on the one hand, they facilitate the organized deposition of collagen fibers, thereby preserving connective tissue architecture and organ function; on the other hand, fibroblasts differentiated from mesenchymal stem cells (MSCs) can become excessively activated by platelet-derived growth factor (PDGF) signaling, thereby inducing pathological collagen deposition and resulting in fibrotic scar formation. This dynamic equilibrium mechanism highlights the biological characteristics of MSCs, which regulate both the repair process and the propensity for fibrosis via the PDGF pathway (Lu et al., 2022).

Fibroblasts, as the principal regulatory cells of the extracellular matrix (ECM), specifically secrete basement membrane glycoprotein family members (NID-1 and NID-2). These highly conserved glycoproteins play a central role in maintaining tissue structural integrity and facilitating ECM remodeling by mediating the structural assembly of collagen fibers and laminin (Pérez-Díaz et al., 2022). Nidogen family members exhibit significant functional heterogeneity. NID-1, a ubiquitous component of the basement membrane, contributes to structural stability via integrin signaling. In skeletal muscle, intramuscular overexpression of NID-1 results in fibrotic deposition. This finding indicates that regulation of NID-1 levels is essential for preventing fibrosis (Pérez-Díaz et al., 2022). In contrast, NID-2 displays a spatially specific expression pattern, particularly during the development of neuromuscular junctions, and preserves the efficiency of neuromuscular signaling by regulating the distribution of synapse-associated proteins (e.g., agrin) (Fox et al., 2008).

#### 3.4.5 MSCs and skeletal muscle repair completion - fibrous scar formation

Fibrous scarring primarily results from the pathological activation of fibroblasts, while the pathological differentiation of MSCs into fibroblasts under inflammatory conditions further increases their number. During tissue repair and regeneration, the inflammatory response functions as the initial mechanism that activates multiple repair pathways. Damaged tissue recruits MSCs to the injury site by secreting chemokines, which subsequently induce their differentiation into fibroblasts within the local microenvironment (Kim and Braun, 2020). In an inflammatory environment, MSCs release large quantities of factors that robustly activate the Transforming Growth Factor-β (TGF-β) signaling pathway. TGF-β then converts quiescent myofibroblasts into an activated state with contractile function through a Smad2/3-dependent pathway, thereby dving the phased remodeling of the extracellular matrix (ECM) to facilitate tissue repair (Rockey et al., 2015). However, sustained activation of TGF-β signaling results in dysregulation of the balance between ECM synthesis and degradation, leading to abnormal collagen fiber deposition that subsequently progresses into pathological fibrotic conditions, including cardiac fibrosis, liver cirrhosis, and skeletal muscle fibrosis (Iyer et al., 2020). This ECM homeostatic imbalance not only results in irreversible dysfunction but also compromises tissue structural integrity through mechanotransduction (Plikus et al., 2021; Larouche et al., 2023). It is noteworthy that the formation of intramuscular fibrotic scars not only signifies the completion of repair but also initiates subsequent regenerative impairment.

Fibrotic scar formation after skeletal muscle injury is known to significantly impair muscle function, whereas transplantation of MSCs has been demonstrated to enhance regenerative outcomes by promoting neovascularization and the myogenic differentiation of stem cells (Linard et al., 2018). The therapeutic effect is primarily attributed to the paracrine activity of MSCs: secreted extracellular vesicles have been shown to promote myogenesis by activating the Wnt/β-catenin signaling pathway in satellite cells, while concurrently inhibiting the TGF-β/Smad3 pathway to attenuate inflammation and fibrosis, thereby facilitating the repair of myofibrillar progenitor fibers and the functional reconstruction of muscle (Bittel and Jaiswal, 2019).

### 3.5 Skeletal muscle damage induced by MSCs depletion

Although abnormal differentiation of myogenic MSCs contributes to an increase in fibroblasts and adipocytes under pathological conditions, the depletion of these cells nonetheless severely disrupts skeletal muscle homeostasis. It has been demonstrated that short-term depletion of MSCs significantly reduces skeletal muscle mass and impairs functional grip strength (Uezumi et al., 2021). Evidence suggests that ablation of fibro/adipogenic progenitors (FAPs) compromises skeletal muscle function: Cre-dependent, diphtheria toxin-mediated FAP depletion leads to impaired expansion of muscle stem cells (MuSCs) and reduced regeneration following injury; under homeostatic conditions, FAP depletion causes muscle atrophy and a decline in the MuSC pool (Murphy et al., 2011; Farup et al., 2015). Kurosawa et al. demonstrated through spectral tracing that MSCs preserve skeletal muscle homeostasis by dynamically regulating the matrix–myocyte interaction network. Their absence results in impaired myofiber regeneration, innervation abnormalities, and ultimately a collapse of the mechanisms responsible for maintaining muscle mass and functional reconstruction (Uezumi et al., 2021).

## 4 Myasthenia gravis

Myasthenia gravis is a chronic autoimmune disorder, the pathogenesis of which involves multiple factors, including immune dysfunction, abnormalities at the neuromuscular junction, and the production of acetylcholine receptor antibodies. The disorder is characterized by a progressive decline in skeletal muscle strength, frequently accompanied by secondary manifestations, including muscle atrophy and generalized weakness, as the condition advances.

### 4.1 MSCs and myasthenia gravis

MSCs have emerged as a potential therapeutic strategy for autoimmune diseases, such as myasthenia gravis, owing to their unique immunomodulatory capacity (Konen et al., 2022). In experimental autoimmune myasthenia gravis (EAMG) animal models, MSC transplantation not only significantly enhances neuromuscular junction signaling efficiency but also reduces abnormally elevated acetylcholine receptor antibody concentrations, restores the balance of immune cell subpopulations, and confers additional regulatory benefits, thereby providing a theoretical foundation for clinical application (Tang et al., 2024; Sudres et al., 2017; Yu et al., 2010). More strikingly, recent research by Jean-Thomas Vilquin’s team applied MSCs co-cultured with PBMCs to a humanized mouse model of myasthenia gravis and demonstrated that this treatment reduced disease severity by half as early as 2 weeks post-injection (Bayer et al., 2025).

Although the effectiveness of MSCs in the treatment of EAMG has been partially confirmed, their underlying mechanisms of action remain to be elucidated in greater depth. The mechanisms of MSCs in the treatment of MG will be elaborated from the perspectives of immunity, the neuromuscular junction, and acetylcholine.

### 4.2 MSCs modulate immunotherapy for skeletal muscle weakness in MG

Myasthenia gravis (MG), as an autoimmune disease closely associated with splenic immune function, has shown clear therapeutic efficacy from allogeneic mesenchymal stem cell (MSC) transplantation. Previous studies have demonstrated that infusion of allogeneic MSCs can alleviate the pathological progression of MG by restoring the imbalance of T-cell subsets. Nevertheless, it remains unclear whether these beneficial effects are mediated through the regulation of the spleen, which serves as the central immune organ. Of note, research in liver cirrhosis has employed In-oxine labeling to trace infused allogeneic MSCs, and these studies revealed their specific homing properties, thereby suggesting that similar mechanisms might also contribute to the therapeutic effects of MSCs in MG (Gholamrezanezhad et al., 2011)^.^ By the 10th day after transplantation, the concentration of MSCs in the spleen was markedly increased relative to baseline levels, suggesting that their preferential homing to the spleen may contribute to immunomodulation.

In addition to the humoral environment, the skeletal muscle microenvironment of patients with MG is characterized by a chronic inflammatory state. The main manifestations include T- and B-lymphocyte infiltration and the sustained release of pro-inflammatory factors. The inflammatory response represents a critical contributor to the pathogenesis of systemic and ocular MG (Molin et al., 2017). This inflammatory state not only inhibits myogenesis but also drives aberrant differentiation of MSCs into adipocytes via activation of the JNK signaling pathway, ultimately resulting in intramuscular adipose infiltration accompanied by fibrosis (Du et al., 2010). Notably, sustained IL-6-mediated activation of the STAT3 pathway modifies the functional behavior of MSCs, predisposing them to fibrotic processes rather than normal tissue repair (Ren et al., 2022; Malecova et al., 2018).

In this pathological context, the inhibition of muscle regeneration caused by the inflammatory microenvironment was reversed by MSCs through the secretion of anti-inflammatory mediators such as IDO, IL-10, and IL-1RA, which synergistically downregulated TNF-α and IL-6 while up-regulating pro-repair factors such as IGF-1 (Ji et al., 2015). This process effectively mitigated persistent attacks induced by immune complex deposition at neuromuscular junctions (Kanatli et al., 2017; Gradolatto et al., 2014). IDO is a hydrolytic enzyme related to tryptophan, and T cell activation primarily requires tryptophan. Moreover, MSCs eliminate activated T cells through the tryptophan metabolic pathway, which synergizes with factors such as ProstaGlandin E2 (PGE2) and TGF-β to remodel immune homeostasis (Ren et al., 2009).

Therapeutic efficacy is exerted by heterologous MSC infusion through a multidimensional immunomodulatory network (Pistoia and Raffaghello, 2017): 1 aberrant T cell activation is inhibited, and regulatory T cell (Treg) differentiation is selectively induced; 2 CD4^+^T cell-mediated B-cell activation and plasma cell differentiation are blocked (Maccario et al., 2005), while the expression of the critical B-cell activation factor BAFF is downregulated, thereby preventing autoantibody production pathways and ameliorating MG pathological progression (Sudres et al., 2017); 3 tissue repair is promoted through the secretion of IL-33, which recruits Tregs to the site of injury (Kuswanto et al., 2016). 4 Pro-inflammatory factors such as IFN-γ, TNF-α, IL-1β can evoke MSCs to modulate the infiltrating T cells by creating nitric oxide (NO)-mediated immunosuppressive microenvironment. This is achieved through inducing high expression of chemokines and iNOS (Ren et al., 2008).

This multidimensional regulatory mechanism enables MSCs to maintain immune homeostasis in the skeletal muscle of patients with myasthenia gravis (MG). On the one hand, MSCs attenuate muscle fiber damage by suppressing excessive immune responses; on the other hand, they promote myofibrillar repair by activating satellite cell function. Such a unique dual effect—combining anti-inflammatory and pro-regenerative properties—provides a theoretical foundation for alleviating skeletal muscle weakness and delaying secondary pathological manifestations in MG.

### 4.3 MSCs act at the neuromuscular junction to treat skeletal muscle weakness in MG

The core pathological feature of myasthenia gravis (MG) is skeletal muscle weakness caused by impaired signal transmission at the neuromuscular junction. Skeletal muscle tissue is composed of highly differentiated multinucleated muscle fibers, and its contractile function is dependent on signaling at the NeuroMuscular Junction (NMJ) - Acetylcholine (ACh) released by motor neurons reaches the muscle membrane through diffusion, and the transmitter triggers action potential conduction by binding to membrane-specific receptors, ultimately triggering the myofilaments to contract. This sophisticated regulatory system ensures the precise response of skeletal muscle to neural commands (Lo et al., 2020).

Modern studies have demonstrated that MSCs play a crucial role in neuromuscular junction reconstruction and nerve regeneration (Kim et al., 2012). Using an Amyotrophic Lateral Sclerosis (ALS) model, Krakora’s team demonstrated that intramuscularly transplanted MSCs release glial cell-derived neurotrophic factor (GDNF) in response to stimulation by the inflammatory microenvironment. As a critical regulator of pre- and postsynaptic differentiation, GDNF has been shown to significantly increase neuromuscular junction density and enhance motor neuron survival by preserving synaptic plasticity (Krakora et al., 2013). In skeletal muscle injury models, transplantation of MSCs has been shown to accelerate repair by reducing collagen deposition, upregulating myogenic marker expression, and promoting neuromuscular junction regeneration. In addition, Ciliary Neurotrophic Factor (CNTF) and Brain-Derived Neurotrophic Factor (BDNF) secreted by MSCs synergistically exert neuroprotective effects: CNTF specifically promotes motor neuron survival and axon regeneration, while BDNF optimizes neural signaling by modulating synaptic plasticity (Ropper et al., 2017).

Chronic impairment of neuromuscular junction transmission is often followed by secondary denervation, which results in the progressive worsening of skeletal muscle atrophy. The characteristic alterations are typified by a reduction in the cross-sectional area of both type I and type II muscle fibers (Snijders et al., 2014). Satellite cells, which represent the primary cellular population responsible for skeletal muscle regeneration, are activated upon injury and subsequently undergo a regenerative cascade involving proliferation, differentiation, and fusion, ultimately contributing to myofiber repair through the incorporation of nascent myonuclei (Chargé and Rudnicki, 2004).

Skeletal muscle atrophy is inhibited by MSCs through the regulation of satellite cell activity and the suppression of myonuclear apoptosis via a paracrine mechanism. Homeostasis of the satellite cell pool is maintained by MSCs primarily through the downregulation of the pro-apoptotic protein Bax, while concurrently up-regulating the anti-apoptotic protein Bcl-2 and activating the phosphorylated Akt (p-Akt) signaling pathway, thereby effectively counteracting muscle wasting (Li et al., 2016). Notably, MSCs and their exosomes (MSC-EXO) alleviate the dysregulation of protein metabolism in skeletal muscle atrophy through activation of the AMPK/ULK1-mediated autophagy pathway (Perry et al., 2016). Mechanistic studies have demonstrated that transplantation of human umbilical cord–derived mesenchymal stem cells (hucMSCs) enhances autophagic activity by activating the AMPK/ULK1 signaling pathway and specifically downregulating the expression of Atrogin-1 and MuRF1, thereby effectively suppressing the pathological progression of skeletal muscle atrophy (Song et al., 2023).

### 4.4 Acetylcholine secretion by MSCs and MG muscle weakness: a potential role

The core pathological mechanism of myasthenia gravis (MG), an autoimmune disease, is closely associated with immune-mediated attack on the acetylcholine receptor (AChR). The pathogenesis involves two principal mechanisms: 1 competitive binding of autoantibodies to the AChR, which interferes with neuromuscular transmission, and 2 antibody-mediated endocytic degradation of AChRs, resulting in reduced receptor density at the postsynaptic membrane. Notably, disease severity is more directly determined by the extent of reduced AChR expression at the muscle endplate than by circulating antibody levels alone (Sudres et al., 2017). Current therapeutic approaches primarily aim to increase acetylcholine concentrations within the synaptic cleft and suppress autoantibody production, for example, by delaying acetylcholine degradation with anticholinesterase agents.

It has been demonstrated that MSCs regulate the cholinergic system through multiple mechanisms. Acetylcholine exerts various physiological effects through muscarinic receptors (mAChRs) and nicotinic receptors (nAChRs), the latter being specifically responsible for regulating skeletal muscle contraction. MSCs promote acetylcholine synthesis and release by upregulating Choline Acetyltransferase (ChAT) and Vesicular Acetylcholine Transporter (VAChT) expression. Concomitantly, the secretion of Prostaglandin E2 (PGE2) activates relevant signaling pathways, thereby enhancing ACh synthesis and release (Zhang et al., 2022). Undifferentiated MSCs inherently possess the capacity to store acetylcholine, which provides the fundamental basis for their regulation of neuromuscular function (Hoogduijn et al., 2006).

Literature studies have demonstrated that, in animal models of MG, the injection of MSCs significantly reduces serum anti-acetylcholine receptor antibody (AChR-Ab) levels (Yu et al., 2010) and restores AChR density at motor endplates (Sudres et al., 2017). On the basis of these findings, it is hypothesized that MSCs may alleviate MG symptoms by enhancing acetylcholine (ACh) synthesis and release, thereby competitively inhibiting the pathogenic effects of circulating AChR-Ab. This proposed mechanism requires further experimental validation.

### 4.5 Senescent MSCs and MG

Aging-related skeletal muscle degeneration is accompanied by increased ectopic fat infiltration and fibrosis (Marcus et al., 2010; Uezumi et al., 2014; Chen et al., 2022), and this pathological process is closely associated with the senescence of MSCs. Senescent MSCs are characterized by reduced autophagy, diminished proliferative capacity, and enhanced lipogenic differentiation, which result in abnormal intramuscular fat accumulation and an imbalance in matrix remodeling (Hansen et al., 2018; Ma et al., 2018). It has been demonstrated that young MSCs preserve stem cell properties under microenvironmental stress via autophagy, whereas senescent MSCs exhibit dysfunction of the autophagy–lysosome system, which induces the accumulation of reactive oxygen species and aberrant activation of p53 signaling, ultimately leading to the collapse of musculoskeletal homeostasis (Ma et al., 2018).

The senescence of MSCs has been shown to mediate the disruption of skeletal muscle homeostasis through key molecular pathways. Bone morphogenetic protein 3b (Bmp3b), an essential factor for maintaining skeletal muscle mass and stabilizing the neuromuscular junction (NMJ), is abundantly expressed in young MSCs but becomes progressively downregulated with aging (Uezumi et al., 2021; Kurosawa et al., 2021). This factor has been reported to delay the progression of sarcopenia by suppressing aberrant differentiation of MSCs (Uezumi et al., 2021). In addition, extracellular vesicles (EVs) secreted by senescent bone marrow-derived MSCs have been shown to suppress the myogenic differentiation of satellite cells, thereby leading to impaired myotube fusion and regenerative failure (Dai et al., 2022).

Late-onset MG shows a high incidence among elderly individuals aged 70–74 years. We speculate that its development may be linked to senescent MSCs; nevertheless, this possibility needs to be substantiated by large-scale clinical studies. We intend to explore this direction in future studies.

### 4.6 Clinical trials of MSC therapy for neuromuscular diseases

Currently, clinical research on MSCs for neuromuscular diseases has provided considerable evidence; however, findings from registered clinical trials in myasthenia gravis (MG) have not yet been disclosed, thereby underscoring a notable research gap.

In comparison, clinical investigations of MSCs in other neuromuscular disorders have made gradual progress: ① Amyotrophic Lateral Sclerosis (ALS): Genetically modified MSCs engineered to secrete neurotrophic factors, when administered intrathecally, have been shown to significantly attenuate the decline in the revised ALS Functional Rating Scale (ALSFRS-R) score and to prolong progression-free survival by approximately 6 months (Petrou et al., 2016). ②Multiple Sclerosis (MS): The combined administration of intravenous and intrathecal infusions of autologous MSCs has been shown to significantly reduce the annualized relapse rate in patients with active-progressive MS and to improve motor function (Petrou et al., 2020).

Although a comprehensive clinical evidence system for MSC-based therapy in MG has not yet been established, a series of clinical trials in ALS and MS has produced a relatively complete body of safety and efficacy evidence. This evidence has provided a solid methodological and biological foundation for translational research on MSCs in neuromuscular diseases such as MG, while suggesting their potential therapeutic value.

## 5 Strategies to enhance the efficiency of MSC treatment

According to data from ClinicalTrials.gov, as of August 2025, more than 1,800 clinical trials involving MSCs have been registered worldwide; however, studies specifically focusing on MG remain scarce. Although several companies are actively promoting the commercialization of MSC injection therapies, the clinical outcomes of translation have generally been substantially lower than those observed in *in vitro* experiments. This disparity underscores the critical scientific importance of elucidating the mechanisms responsible for the limited efficacy of MSC therapy (Levy et al., 2020). Currently, multiple research teams are dedicated to optimizing MSC function through diverse strategies aimed at enhancing its translational efficiency from basic research to clinical practice.

### 5.1 Basic experiments to enhance the efficiency of MSC treatment

In order to deeply analyze the potential mechanism of MSCs injection therapy, various research teams have revealed the key regulatory elements through multi-dimensional experimental systems. The current consensus is that the therapeutic effects of MSCs are primarily mediated through the following mechanisms: 1 secretion of immunomodulatory factors (e.g., IL-10, TGF-β): that modulate the local inflammatory microenvironment; 2 release of pro-angiogenic factors (VEGF, bFGF) that facilitate the reconstruction of tissue blood circulation; 3 delivery of anti-apoptotic signals (e.g., Bcl-2 family proteins) through exosomes, which protect damaged cells; and 4 genetic or molecular engineering to confer targeted differentiation potential or specific protein expression capacity. These findings provide a theoretical foundation for the development of next-generation therapies based on precision-engineered MSCs.

Regarding the mechanism of action of transplanted MSCs *in vivo*, several perspectives have been proposed: it has been suggested that transplanted MSCs primarily function through immunomodulation rather than direct tissue regeneration (Hoang et al., 2022). Another perspective suggests that although most exogenous MSCs undergo apoptosis, a subset of transplanted MSCs may still differentiate into myoblasts (Dezawa et al., 2005). Clinical observations by another researcher indicated that MSC infusion elicited a response only when patients exhibited elevated cytotoxic activity. Therefore, the researcher proposed that *in vitro*–induced apoptotic MSCs might be utilized as a therapeutic strategy. After infusion, recipient phagocytes phagocytose apoptotic MSC and produce indoleamine 2,3-dioxygenase, thus realizing immunosuppression (Galleu et al., 2017).

Optimization of therapeutic parameters is crucial for determining the efficacy of MSCs. Administration of MSCs during the acute phase of injury results in minimal regenerative benefit, whereas administration at the peak of the inflammatory response markedly enhances myofiber regeneration and capillary density (Helal et al., 2016). The improvement in muscle strength is most evident when the graft volume reaches 1.0 × 10^6 cells in rat soleus muscles after severe crush trauma (Qazi et al., 2019).

Hypoxic preconditioning has been shown to enhance the regenerative efficacy of MSCs through a mechanism involving the hypoxia-inducible factor (HIF-1α)-mediated upregulation of VEGF and WNT4, thereby promoting vascularization and myogenesis (Archacka et al., 2021; Zhang et al., 2015).

### 5.2 Selecting sources of MSCs to improve therapeutic efficiency

Bone marrow–derived mesenchymal stem cells (BMMSCs) are constrained by invasive collection procedures, ethical concerns, and challenges in large-scale expansion. Consequently, the pursuit of alternative cell sources has emerged as a prominent research focus. Adipose-derived mesenchymal stem cells (ADSCs) represent a significant breakthrough, as they can be isolated through minimally invasive liposuction. ADSCs have emerged as an ideal progenitor cell source for regenerative medicine, owing to their accessibility (abundant adipose tissue reserves and minimal donor trauma) and robust proliferative capacity (Cannon et al., 2003; Jack et al., 2005). Studies have demonstrated that ADSCs exhibit enhanced myogenic differentiation potential compared with BMMSCs, conferring notable advantages in promoting myofiber neogenesis and structural tissue reconstruction (de la Garza-Rodea et al., 2012). In particular, the transplantation of BMMSCs has been reported to induce abnormal remodeling of the collagen matrix, which results in the formation of pathological fibrotic foci and impaired skeletal muscle contractile function. In contrast, the application of ADSCs has been shown to markedly suppress excessive collagen deposition and promote the restoration of microenvironmental homeostasis (Moussa et al., 2020). However, ADSCs still have technical bottlenecks such as fluctuating phenotypic plasticity and heterogeneity of therapeutic responses, so their potential for clinical application as an alternative to BMMSCs still needs to be carefully evaluated (Mohamed-Ahmed et al., 2021).

Muscle-derived mesenchymal stem cells (mdMSCs) have been recognized as a highly promising therapeutic approach because of their distinct clinical applicability and functional characteristics. Utilizing minimally invasive muscle micro-biopsy techniques, mdMSCs can be obtained in a highly scalable manner, while avoiding the risk of immune rejection and substantially reducing patient trauma, thereby providing greater clinical advantages compared with bone marrow- or adipose tissue-derived MSCs. Functional studies have demonstrated that mdMSCs possess significant angiogenic, immunomodulatory, and anti-fibrotic capacities through paracrine mechanisms. Their secretome is enriched with pro-repair factors, which effectively suppress inflammatory responses and promote angiogenesis in *in vitro* models (Guesdon et al., 2025).

Modern studies have shown that researchers have successfully explored other sources of MSCs for therapeutic application such as umbilical cord/amniotic fluid: extra-embryonic tissue-derived MSCs (e.g., umbilical cord/amniotic fluid) are favored because of their embryonic stem cell-like phenotypic profile and lesser ethical concerns (Kim et al., 2012; Shareghi-Oskoue et al., 2021). Tonsil-derived mesenchymal stem cells (T-MSCs), obtained from surgical waste such as tonsils, have emerged as a focus of regenerative medicine research because of their distinctive immunomodulatory properties and remarkable proliferative capacity (Cho et al., 2015; Park et al., 2019). This innovative cell source offers an ethically compliant, scalable, and high-quality solution to advance mesenchymal stem cell–based therapeutic strategies.

### 5.3 Transforming MSCs with engineering to improve efficiency

Modern bioengineering often incorporates therapeutic molecules (e.g., VEGF, IL-10) into MSCs via viral transduction or gene-editing methods to achieve targeted therapeutic effects (von Bahr et al., 2012). MSCs retain stemness markers (Oct4+, Nanog+) and exhibit robust proliferative capacity, and it has been demonstrated that MSCs cultured over successive passages preserve karyotypic stability and trilineage differentiation potential (Debnath and Chelluri, 2019). This property, together with the CXCR4/SDF-1 axis–mediated homing ability, renders MSCs an ideal biocarrier. However, it should be noted that long-term infusion of allogeneic MSCs may pose a risk of metabolic disorders, as postmortem pathological analyses have revealed an abnormal 32% increase in mitochondrial respiratory chain complex activity (von Bahr et al., 2012).

Previous studies have demonstrated that the primary factor limiting the efficacy of MSCs is closely associated with their survival microenvironment. Following xenotransplantation, MSCs undergo apoptosis in response to alterations in the microenvironment, including changes in extracellular matrix composition, oxygen partial pressure, and nutrient availability, ultimately resulting in a marked reduction in the number of viable cells. To overcome this bottleneck, various engineering strategies have been developed, such as the maintenance of MSC stemness through sulfated alginate encapsulation, which markedly enhances their survival during skeletal muscle regeneration (Gionet-Gonzales et al., 2023). Implantation of an epicardial patch loaded with MSCs on the cardiac surface after coronary artery bypass grafting significantly improves the prognosis of postoperative cardiac function in patients by ameliorating the dysregulation of mitochondrial proteomic profile (Agrawal and Thankam, 2021).

The development of novel delivery systems has provided innovative strategies to optimize the therapeutic efficacy of MSCs. For instance, 3D bioprinting technology has been employed to fabricate cardiac patches that co-encapsulate bone marrow–derived MSCs (BM-MSCs) with genetically engineered MSCs expressing hepatocyte growth factor (HGF). Following implantation into the infarcted myocardial region, this system has been shown to extend MSC survival by more than threefold and to promote both neovascularization and myocardial tissue repair (Park et al., 2020). In addition, the combined application of ultrasound-targeted microbubble disruption (UTMD) technology with platelet-derived growth factor-BB (PDGF-BB) pretreatment has been shown to enhance the homing efficiency of MSCs through upregulation of stromal cell–derived factor-1 (SDF-1) and to inhibit apoptosis of grafted cells, thereby improving the therapeutic efficacy of ischemic myocardial repair (Sun et al., 2023).

At the mechanistic level, proteomic tracking utilizing a mutant methoxy-tRNA synthetase (MetRS^L274G) demonstrated that MSCs transplanted into ischemic hearts underwent extensive proteomic remodeling, encompassing pathways such as the complement–coagulation cascade, fibrinogen activation, regulation of apoptosis, and actin cytoskeleton remodeling. Among them, complement factor H (CFH) and actin depolymerization-associated proteins (e.g., cofilin, fibronectin) play a key role in cardiac repair by regulating cell migration, proliferation, and neovascularization (Han et al., 2020).

## 6 Safety and tumorigenicity of MSCs

Although MSCs exhibit sustained proliferation characteristics similar to those of cancer cells, their tumorigenic risk remains considerably lower than theoretically predicted (Serakinci et al., 2004). To meet the requirements for clinical-scale cell production, extensive expansion is required. Genomic variations have been observed to emerge during serial passages in most hUC-MSC systems. Importantly, no malignant transformation has been reported in these variations to date; however, the establishment of routine genomic monitoring and donor screening mechanisms is strongly recommended prior to clinical application (Wang et al., 2013). Further studies have demonstrated that prolonged culture may induce chromosomal abnormalities, increase tumorigenic risk, impair immunomodulatory function, and reduce homing ability, potentially leading to treatment failure. These findings underscore the necessity of strictly controlling culture duration (Yong et al., 2018).

Cryopreservation is regarded as a primary strategy for preserving early-passage hMSCs, facilitating the creation of ready-to-use cell banks to satisfy clinical requirements. Thawing assays of human adipose-derived MSCs (hASCs) following 3 months of cryostorage revealed stable expression of tumor-suppressor markers (p53, p21, p16, and pRb), maintenance of normal telomerase activity and telomere length, and an absence of significant DNA damage or p53 mutations, thereby confirming that the cryopreservation process does not elevate tumorigenic risk (Yong et al., 2017). A meta-analysis has also provided evidence supporting the clinical safety of MSC therapies (Lalu et al., 2012); however, large-scale controlled trials and standardized adverse-event reporting systems are still required to further substantiate long-term safety benchmarks.

## 7 Limitations and perspectives

This study has certain limitations. Owing to the current stage of development in MG clinical treatment, MSC-based therapies have not yet advanced to registered clinical trials (according to a ClinicalTrials.gov search conducted up to August 2025). As a result, a systematic analysis of the clinical progress, translational pathways, and regulatory frameworks for MSC therapy in MG cannot be performed, thereby limiting the depth of discussion on related translational bottlenecks. The lack of such clinical trial data represents a significant limitation of this study and will remain a critical focus for future research advances.

Nevertheless, the present study focuses on the central objective of preclinical research: analyzing the multi-mechanistic synergistic network by which MSCs promote skeletal muscle regeneration (including paracrine effects, immunomodulation, and angiogenesis) and integrating evidence from animal models to infer potential therapeutic pathways of MSC therapy for MG. This theoretical framework serves as a mechanistic foundation for emerging therapies that lack direct clinical evidence and, by integrating preclinical data from related diseases (such as muscular dystrophy and polymyositis), establishes a scientific basis for designing future first-in-human clinical trials of MSCs for MG. It further highlights prioritized research directions in critical areas, including cell subpopulation selection and optimization of delivery strategies. The primary tasks of subsequent research will involve continuously monitoring the clinical translation of MSC therapy for MG and promptly incorporating emerging clinical trial data to refine the translational pathway model.

In 2025, the International Society for Cellular Therapy (ISCT) revised and reissued the criteria for identifying MSCs, with the primary modification being the redefinition of their functional role. By removing the stemness-related clause in the original standard, which required verification of “osteogenic/lipogenic differentiation,” ISCT, for the first time, established a clear technical distinction between MSCs and embryonic stem cells, characterizing MSCs as primarily immunomodulatory rather than broadly multipotent. This revision was grounded in key clinical findings: in most injectable therapeutic applications, MSCs exert their effects predominantly through paracrine mechanisms (e.g., the release of cytokines, exosomes, and other bioactive substances), rather than by directly differentiating into target tissue cells.

It is worth noting in particular that this revision of the standard does not negate the inherent biological properties of MSCs, but rather establishes a more precise definition system. MSCs from different tissue sources exhibit varied stemness characteristics, especially in specific therapeutic scenarios such as bone tissue repair, where their stemness potential remains an important basis for achieving clinical efficacy. For this reason, the standard explicitly mandates that the tissue source of MSCs be clearly identified and requires the submission of corresponding documents verifying biological properties based on the source type. This dual specification not only effectively prevents ethical controversies associated with embryonic stem cells but also preserves flexibility for the scientific and rational application of MSCs with diverse characteristics.

With respect to the newly released ISCT guidelines, several considerations remain: In the treatment of muscular diseases, the immunomodulatory function of MSCs, although crucial for suppressing inflammatory responses, should not be regarded as the sole mechanism of action. An exclusive emphasis on this single pathway may obscure their synergistic roles in angiogenesis and neural repair, and effective tissue regeneration is fundamentally dependent on the integration of multiple pathways.

The current scientific consensus on the therapeutic mechanism of MSCs is centered on the paracrine-dominant hypothesis, which posits that injected cells primarily facilitate tissue repair through the transient secretion of bioactive factors. This paradigm is largely supported by tracing technologies (e.g., infrared fluorescent labeling), which have demonstrated the clearance of transplanted cells within approximately 3 weeks (Chiu et al., 2020). However, a critical limitation remains: tracing markers can monitor only the initially injected cells and cannot track potential progeny. If a subset of MSCs undergoes mitosis *in vivo*, their differentiation trajectory and long-term biological behavior remain undetectable. Given the robust proliferative capacity of primary BMSCs observed *in vitro* (adherence within 3 days; confluent growth in a 10-cm dish within 10 days), the skeletal muscle microenvironment, although nutrient-restricted, does not entirely rule out the possibility that a small surviving fraction may contribute to muscle regeneration. More critically, if the multipotent differentiation capacity is not verified prior to injection, and if the cells harbor differentiation abnormalities (e.g., genetic mutations or epigenetically altered states induced by culture), they may aberrantly differentiate into fibroblasts or adipocytes within muscle tissue. Such aberrant differentiation could subsequently lead to localized fibrosis or fatty infiltration. Although this risk has not been definitively documented in clinical settings, it may theoretically negate the reparative benefits of paracrine signaling and further exacerbate pathological progression. Consequently, the omission of osteogenic and adipogenic validation requires more stringent *in vivo* safety assessments (e.g., long-term histological monitoring) to balance standardization requirements with inherent biological uncertainties.

Future research may advance in the following directions: 1 the development of engineered MSCs aimed at enhancing targeted homing ability, overexpressing neurotrophic factors (e.g., GDNF, CNTF), or silencing fibrosis-associated pathways (e.g., TGF-β) through gene-editing techniques; 2 the establishment of MG-specific organoid models to simulate the pathological microenvironment, thereby elucidating the interaction networks between MSCs, satellite cells, and immune cells; 3 exploration of strategies for the standardized preparation and functional optimization of MSC-derived exosomes, in combination with single-cell sequencing technology to identify key active components; and 4 interdisciplinary research integrating MSC therapy with antifibrotic drugs and immunosuppressants to establish a multimodal intervention system. In addition, the development of artificial intelligence–based efficacy prediction models, together with the accumulation of long-term follow-up data, is expected to accelerate clinical translation and provide precise guidance for individualized treatment.

## 8 Conclusion

The present study demonstrates that MSCs exhibit unique advantages in the treatment of MG and muscle degenerative diseases through multidimensional mechanisms: they can remodel the immune homeostasis of the neuromuscular junction through immunomodulation (e.g., inhibition of BAFF/TNF-α signaling, inducing Treg differentiation); they also secrete neurotrophic factors (CNTF, BDNF) to promote axon regeneration and synaptic plasticity; and they can promote acetylcholine secretion. In aging muscle lesions, MSCs are reported to maintain stem cell homeostasis by regulating Bmp3b expression, to inhibit ectopic fat deposition and fibrosis progression, and to restore protein metabolic balance through activation of the AMPK/ULK1 autophagy pathway. However, senescence of MSCs results in functional decline and increased dependence on the microenvironment, indicating the necessity of further optimization in application strategies. Future investigations should prioritize the development of engineered MSCs, strategies for senescent cell clearance, and exosome-based targeted delivery systems, in order to achieve comprehensive therapeutic coverage from pathological mechanisms to functional reconstruction, thereby establishing a new paradigm for precision medicine in neuromuscular disorders.

Based on a comprehensive evaluation of the application potential of various mesenchymal stem cells, it can be concluded that MSCs from different sources possess distinct advantages and specific clinical applications. Bone marrow-derived MSCs (BMMSCs), being the most extensively studied type, exhibit well-characterized biological properties. However, their clinical application has gradually declined owing to the invasiveness of harvesting methods and the associated risk of fibrosis. Adipose-derived MSCs (ADSCs) present notable advantages in muscle regeneration because of their accessibility, robust proliferative capacity, and remarkable myogenic differentiation potential. Nevertheless, functional heterogeneity restricts their therapeutic consistency. Umbilical cord-derived MSCs (UC-MSCs), by contrast, exhibit distinctive and comprehensive advantages: their collection is non-invasive and ethically acceptable, they display robust proliferative capacity and multidirectional differentiation potential, and their low immunogenicity renders them particularly suitable for large-scale production and allogeneic transplantation. Although existing studies have demonstrated that UC-MSCs possess significant potential in angiogenesis and tissue repair, their ability for directed differentiation into specific tissues (e.g., muscle) remains insufficiently investigated. Therefore, the selection of the most appropriate MSC source should be determined by specific therapeutic requirements: ADSCs are likely to be more suitable for muscle-specific regeneration, whereas UC-MSCs appear to hold broader clinical prospects in contexts that demand large-scale, standardized cell therapies.
